# The association of dietary patterns and adherence to WHO healthy diet with metabolic syndrome in children and adolescents: Tehran lipid and glucose study

**DOI:** 10.1186/s12889-019-7779-9

**Published:** 2019-11-06

**Authors:** Parvin Mirmiran, Maryam Ziadlou, Sara Karimi, Firoozeh Hosseini-Esfahani, Fereidoun Azizi

**Affiliations:** 1 Nutrition and Endocrine Research Center, Research Institute for Endocrine Sciences, Shahid Beheshti University of Medical Sciences, Tehran, Iran; 2 Endocrine Research Center, Research Institute for Endocrine Sciences, Shahid Beheshti University of Medical Sciences, Tehran, Iran

**Keywords:** Dietary pattern, Healthy diet, World health organization, Metabolic syndrome

## Abstract

**Background:**

The optimal dietary pattern for reducing the extent of metabolic syndrome (MetS) has not been well established yet. The aim of this study was to evaluate dietary patterns and adherence to WHO healthy diet in children and adolescents and their associations with MetS.

**Methods:**

Subjects of this cohort study were selected from among children and adolescents of the Tehran Lipid and Glucose Study participants, aged 6–18 years (*n* = 424). Dietary measurements were collected using a valid and reliable food frequency questionnaire. MetS was defined as the existence of at least 3 risk factors according to the Cook criteria. Diet was assessed based on dietary components of the WHO healthy diet. Dietary patterns were defined by principal component analysis.

**Results:**

The mean ± SD age of participants (42% boys and 57% girls) was 13.5 ± 3.7 years. The most consistency with the WHO healthy diet was observed for cholesterol, free sugar and protein consumption in both genders, and the least was for n-3 poly-unsaturated fatty acid, trans-fatty acid and salt. Intake of SFA up to 12% of energy intake (third quartile) reduced the risk of MetS, compared to the first quartile. Subjects in the third quartile of n-6 poly-unsaturated fatty acid intake (6.2% of energy) showed the lowest odds ratio of MetS compared to the first quartile (OR: 0.18, CI: 0.04–0.66). In the adjusted model, the risk of MetS reduced across quartiles of MUFA intake by 60% (OR: 1, 0.40, 0.40, 0.42; P trend = 0.05). No significant trends were observed in the risk of MetS components across quartiles of the WHO healthy diet components. Three major dietary patterns were identified, the healthy, unhealthy and cereal/meat. An increased risk of MetS was observed in the highest quartile of unhealthy dietary pattern score compared to the lowest quartile (OR: 1, 0.81, 0.93, 2.49; P trend = 0.03).

**Conclusions:**

Our results demonstrated that the majority of our population did not meet some components of WHO healthy diet recommendations. The quality and quantity of fatty acid intakes were associated with risk of MetS. Adherence to unhealthy dietary pattern was associated with two-fold increase in MetS risk.

## Background

A collection of multifactorial metabolic disorders, including abdominal obesity, dyslipidemia, hypertension and hyperglycemia is called Metabolic Syndrome (MetS) [1]. The prevalence of MetS is increasing in children and adolescents of many countries, including Iran [2, 3]. MetS has been associated with a higher risk of cardiovascular disease and type 2 diabetes in adulthood, thus, becoming a major public health concern worldwide [4]. Diet plays a very important role in the growth and development of adolescents, during which the formation of healthy eating habits is important [5]. Unhealthy dietary patterns and lifestyle habits established during childhood and adolescence are significantly tracked into adulthood [6]. Dietary pattern is one of the risk factors reported to have a significant association with MetS and its related metabolic disorders [7]; however, the optimal local dietary guidelines for reducing MetS has not been well established yet [3]. Also dietary habits are largely influenced by nutrition transition which taking place in Iran in the context of demographic and socioeconomic changes. People are now consuming more foods high in energy, fats, free sugars or salt/sodium, and many do not eat enough dietary fibers including fruit, vegetable and whole grains [8]. Dietary saturated fatty acids (SFA) were positively associated with the prevalence of MetS in Iranian adults, independent of total dietary fat, mono-unsaturated fatty acid (MUFA) and poly-unsaturated fatty acid (PUFA) intakes [9]. Energy-dense nutrient-poor snacks, both salty and sweet, had undesirable effects on the incidence of MetS in Iranian children and adolescents [7, 10]. The World Health Organization (WHO) has issued dietary recommendations and guidelines on nutrition to help reduce the risk of chronic diseases and promote good health [11, 12], one of which is the Healthy Diet fact sheet [8] that includes practical advices on maintaining a healthy diet; recommending a shift in fat consumption away from SFAs to un-saturated fats and limiting total fat to less than 30% of total energy intake. Also this factsheet indicates elimination of free sugars to less than 10% of total energy intake and industrial trans-fats. A further reduction of free sugars to less than 5% of total energy intake is suggested for additional health benefits; moreover, reducing salt intake to less than 5 g per day is recommended for preventing hypertension and reducing the risk of heart disease and stroke in the adult population [8]. To our knowledge, limited data are available on the association of WHO healthy diet with metabolic abnormalities [7, 10]. In this study, we aimed to investigate the association of dietary patterns and adherence to WHO healthy diet and their association with MetS in Iranian children and adolescents of 6–18 years during 3.6 years of follow up.

## Methods

### Subjects

This population-based cohort study was done in the context of the Tehran Lipid and Glucose Study (TLGS), on residents of district no.13 of Tehran, the capital of Iran. These subjects, aged ≥3 years, were chosen by multistage cluster sampling, whose age and socio-economic status are illustrative of Tehran’s overall population [13]. This study aimed to determine the prevalence of non-communicable disease risk factors. The first survey was initiated in 1999–2001, and the second (2002–2005), the third (2006–2008), and the fourth (2009–2011) were follow-up surveys.

**Fig. 1:**
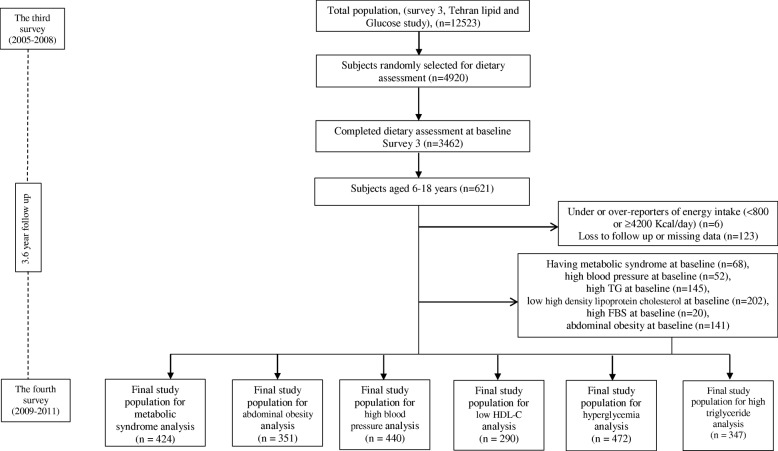
Outline of study participants’ selection

Of total subjects who entered in the third survey (*n* = 12,523), 3462 subjects were chosen randomly for dietary assessment; including 621 children and adolescents aged 6–18 years. Participants were followed for a mean of 3.6 years at the fourth examination survey to evaluate the development of MetS and its components. Subjects who were lost to follow up (*n* = 123) (response rate: 80%) and those with energy intake < 800 or ≥ 4200 kcal/day (*n* = 6) were excluded. Those who had MetS (*n* = 68), hypertension (*n* = 52), high triglycerides (TGs) (*n* = 145), low high density lipoprotein cholesterol (HDL-C) (*n* = 202), high fasting blood sugar (FBS) (n = 20), or abdominal obesity (*n* = 141) at baseline were also excluded for individual analysis of MetS and its components’ incidence. The final sample sizes varied by outcomes as follows: MetS (*n* = 424), abdominal obesity (*n* = 351), high TGs (*n* = 347), hypertension (*n* = 440), low HDL-C (*n* = 290), and high FBS (*n* = 472) (Fig. 1).

### Measurements

Weight was measured, using digital scales, and recorded to the nearest 100 g (Seca 707; Seca Corporation, Hanover, Maryland; range, 0.1–150) kg while participants were minimally clothed without shoes. Height was measured to the nearest o.1 cm using a tape meter (model 208 Portable Body Meter Measuring Device; Seca) with subjects in standing position and without shoes. Body mass index (BMI) was calculated as weight (kg) divided by square of height (m^2^).

Waist circumference (WC) was recorded to the nearest 0.1 cm, using a measuring tape at the umbilicus and without any pressure on body surface. The anthropometric measurement were carried out by one examiner for women and one for men to avoid subjective errors.

Before measuring blood pressure (BP), the participants remained seated for 15 min, and then using a standard mercury sphygmomanometer with the cuff placed on the right arm, blood pressure was measured twice by a clinician and mean values were documented [13].

To measure blood glucose and lipid levels including TGs and HDL-C, blood samples, were obtained by a trained staff, from all subjects at baseline and follow-up, between 7 and 9 am after 12–14 h of fasting. All tests were done at the TLGS research laboratory on the day of blood collection. The enzymatic colorimetric method was used for FBS measurement. Serum concentration of TGs was estimated using commercially available enzymatic reagent (Pars Azmoon, Tehran, Iran) adapted to the Selectra auto analyzer. HDL-C was estimated after precipitation of the apolipoprotein B with a solution of phosphotungstic acid. Inter- and intra assay coefficients of variations were both 2.2% for FBS, 2.0 and 0.5% for HDL-C and 1.6 and 0.6% for TGs, respectively [2].

### Dietary assessment

Dietary intakes were collected by trained research nutritionist, using a valid and reliable 168-items semi-quantitative food frequency questionnaire (FFQ) [14, 15]. Participants were asked (face to face), to report their consumption frequency during the previous year; when children were unable to recall, their mothers were asked about the type and quantity of meals and snacks. Portion sizes of food intakes, reported in household measures, were converted to daily intakes (gr/day). As the Iranian food composition table (FCT) is incomplete, the US Department of Agriculture FCT was used for analyzing energy and nutrient contents; for traditional foods not listed in the US Department of Agriculture FCT, the Iranian FCT was used [16, 17].

### Physical activity assessment

Information on physical activity was collected using the modifiable activity questionnaire (MAQ); high reliability (97%) and moderate validity (49%) have been ascertained previously for the Persian translated MAQ in adolescents. Metabolic equivalent (MET) task (minutes per week) was calculated [18, 19].

### Definitions

#### Metabolic syndrome (MetS)

In children and adolescents, Cook’s criteria was used for definition of MetS, proposed as having ≥3 of the following [20]: fasting TGs ≥110 mg/dl; HDL-C < 40 mg/dl; WC ≥ 90th percentile for age and sex, based on the national reference curves [21]; systolic BP and diastolic BP ≥ 90th percentile for sex, age and height, according to cut points of the National Heart, Lung and Blood Institute [22], and FPG ≥ 100 mg/dl, based on the recommendations of American Diabetes Association.

#### Nutrient intake goals of the WHO healthy diet

The components of the WHO healthy diet include taking cholesterol < 300 mg per day, total fat less than 30%, SFAs less than 10%, and trans-fatty acids (TFAs) less than 1% of total energy intake, and replacing them with unsaturated fats including PUFAs (6–10% of total energy intake), n-6 PUFA (5–8% of total energy intake) and n-3 PUFA (1–2% of total energy intake); also, eating at least 400 g or 5 portions of fruits and vegetables per day to prevent non-communicable diseases and help ensure an adequate intake of dietary fiber (> 25 g). Consuming protein 10–15%, carbohydrates 55–75% and free sugars less than 10% of total energy and salt less than 5 g are other parts of WHO healthy diet [8]. Participants who met the component of the WHO healthy diet recommendations are complier of that component.

### Statistical analyses

**Fig. 2:**
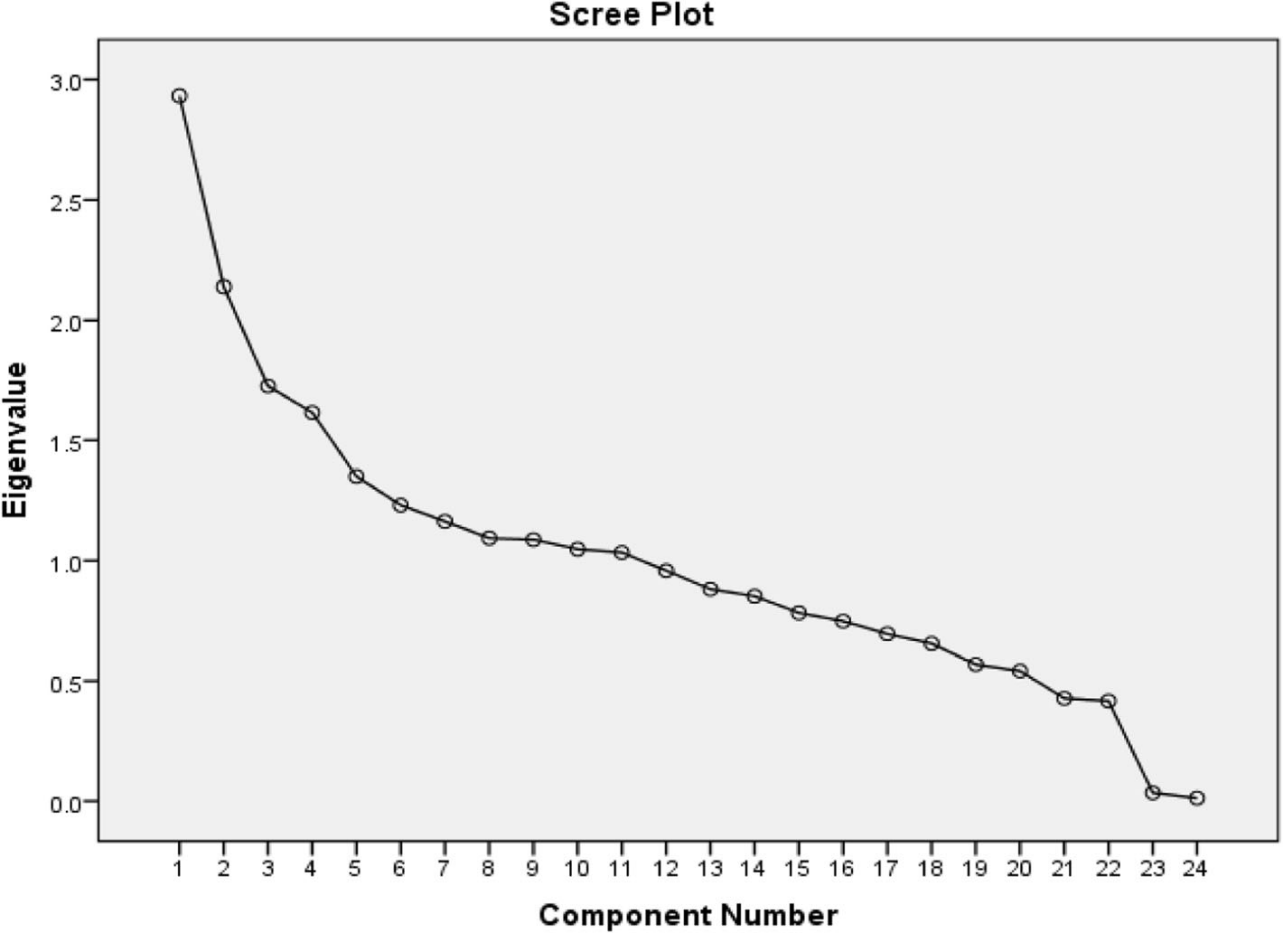
The scree-plot of principal component analysis for extracting major dietary patterns

For analyzing data, the SPSS V.20 (SPSS Inc., Chicago, Illinois) was used. Continues variables were reported as mean ± SD and the categorical as percentages. A comparison of qualitative and quantitative variables among MetS and non-MetS subjects was done using the student T and Chi-square tests, respectively. Dietary patterns were determined using factor analysis with varimax rotation, based on 21 food groups. The patterns were extracted based on the eigenvalues (> 1), scree plot and factor interpretability (Fig. 2). Logistic regression analysis was used to estimate the odds ratio (OR) of MetS according to quartiles of WHO healthy diet components and dietary pattern scores after applying an adjustment for baseline age, sex, energy intake, family history of diabetes (yes or no) and BMI. Physical activity level (continuous) did not change the results, so this variable was excluded from the model. *P* value for trend was determined by logistic regression models using the median of each quartile of dietary factors as a continuous variable.

## Results

The mean ± SD age of participants (42% boys and 57% girls) was 13.5 ± 3.71 years. There was no significant difference in physical activity level between MetS and non-MetS subjects. Also WC and TG level of MetS individuals were higher than non-MetS subjects. HDL-C level was lower in MetS than non-MetS subjects (Table 1).

**Table 1:** Baseline characteristics of children and adolescents (6–19 years) participating in the Tehran Lipid and Glucose Study, 2005–8

	Total (*n* = 424)	Non-MetS subjects (*n* = 376)	MetS subjects (*n* = 47)	*P* value ^†^
Age (year)	13.5 ± 3.71^*^	13.7 ± 3.7	12.7 ± 3.6	0.10
Family history of diabetes (%)	5.2	5.6	2.2	0.51
Waist circumference (cm)	68.8 ± 10.4	68.0 ± 10.1	75.5 ± 11.2	< 0.01
Systolic blood pressure (mmHg)	97.9 ± 11.9	97.6 ± 11.8	101 ± 10.2	0.07
Diastolic blood pressure (mmHg)	64.3 ± 10.0	64.5 ± 9.7	65.3 ± 9.5	0.64
Fasting plasma glucose (mg/dl)	84.9 ± 6.15	84.7 ± 6.12	87.2 ± 5.9	0.008
High density lipoprotein cholesterol (mg/dl)	45.8 ± 10.5	46.5 ± 10.8	40.7 ± 6.8	< 0.01
Triglycerides (mg/dl)	89.1 ± 42.2	79.0 ± 1.4	106 ± 1.5	< 0.01
Physical activity (Met/hour/week)	14.9 ± 14.7	15.1 ± 15.4	13.3 ± 7.8	0.41

^*^ Values are reported as mean ± SD or percentages, using independent t-test for quantitative variables and chi square for qualitative variables

^†^ *P* value: shown as difference between MetS and non-MetS subjects at baseline

The cumulative incidence of MetS was 11.1% after 3.6 years of follow up.

Table 2 shows the percentage of children and adolescents complying with WHO Healthy Diet by sex. The highest compliance was for cholesterol (100%), free sugar (> 80%) and protein intake (> 75%) in both genders, and the least was observed for n-3 PUFAs (< 2%), TFAs (< 7%) and salt (< 16%), without a significant difference between boys and girls. In this population, the mean intake of total fat, SFAs and TFAs were higher than WHO recommendations; the most inconsistency was for TFAs which was received 2 fold higher than recommended. The mean intakes of fruits and vegetables were higher than WHO Healthy Diet recommendations, and the compliance of fruit and vegetable with WHO was > 60% in both genders.

**Table 2:** Dietary intake and percentage of children and adolescents complying with WHO healthy diet in the Tehran Lipid and Glucose Study

Dietary intakes	Recommendation	Compliers^*^_(%)_		Intake _(mean ± SD)_		P^**^
		boys	girls	boys	girls	
Total fat _(% of Energy)_	15–30	42	36.6	32.1 ± 6.38	32.4 ± 7.39	0.71
SFA _(% of Energy)_	< 10	34.3	41.2	11.2 ± 3.06	11.0 ± 3.07	0.38
PUFAs _(% of Energy)_	6–10	51.9	50.6	6.52 ± 1.99	6.68 ± 2.40	0.47
n-6 PUFAs _(% of Energy)_	5–8	48.6	49	5.73 ± 1.88	5.83 ± 2.23	0.61
n-3 PUFAs _(% of Energy)_	1–2	1.1	1.2	0.49 ± 0.17	0.52 ± 0.32	0.34
Trans fatty acid _(% of Energy)_	< 1	6.1	5.8	2.26 ± 1.03	2.20 ± 1.18	0.54
Cholesterol _(mg/day)_	< 300	100	100	151 ± 26.4	154 ± 27.4	0.25
Total carbohydrate (% of Energy)	55–75	65.7	60.5	57.2 ± 6.58	56.8 ± 7.76	0.60
Fruits & vegetables _(g/day)_	≥400	64.6	67.1	606 ± 429	595 ± 361	0.75
Total dietary fiber (g/day)	> 25	46.4	42.4	27.5 ± 15.2	25.3 ± 13.1	0.10
Free sugars _(% of Energy)_	< 10	81.2	84.4	7.05 ± 3.93	6.84 ± 4.74	0.62
Protein _(% of Energy)_	10–15	78.5	76.1	12.9 ± 2.07	13.2 ± 2.37	0.16
Salt _(g/day)_	< 5	12.2	15.2	11.9 ± 7.69	12.2 ± 9.9	0.32

SFA: Saturated fatty acid, PUFA: Poly unsaturated fatty acids

^*^Participants who met each component of the WHO healthy diet recommendations are complier of that component

^**^P value: shown as difference of dietary intakes between genders using t-test

The risk of MetS incidence according to quartiles of WHO healthy diet components is shown in Table 3**.** In the adjusted model, intake of SFA up to 12% of energy intake (third quartile) reduced the risk of MetS, compared to the first quartile. In the crude model, the risk of MetS reduced across quartiles (Q) of MUFA intake (ORs from Q1 to Q4: 1, 0.62, 0.39, 0.45; P trend = 0.03); in the adjusted model, the risk of MetS decreased by 60% (ORs from Q1 to Q4: 1, 0.40, 0.40, 0.42; P trend = 0.05). In both crude and adjusted models, subjects in the third quartile of n-6 PUFA intake (6.2% of energy) showed the lowest odds ratio of MetS compared to the first quartile (OR for Q3: 0.18, CI: 0.04–0.66). No significant trends were observed in the risk of MetS components across quartiles of the WHO healthy diet components.

**Table 3:** Multivariable-adjusted odds ratio (95% CIs) for incident MetS according to quartiles of WHO healthy diet components among 424 children and adolescents

	Dietary intakes				
	Q1	Q2	Q3	Q4	P _trend_
**Total fat** (% of Energy)	24.7	29.8	34.3	39.7	
Model 1	1.00	0.60 (0.26–1.36)	0.54 (0.23–1.25)	0.48 (0.20–1.14)	0.86
Model 2	1.00	0.51 (0.21–1.24)	0.51 (0.20–1.28)	0.40 (0.15–1.05)	0.06
SFA (% of Energy)	7.61	10.0	11.8	14.7	
Model 1	1.00	0.30(0.12–0.75)	0.21(0.07–0.59)	0.70(0.34–1.47)	0.34
Model 2	1.00	0.26(0.09–0.37)	0.18(0.06–0.55)	0.57(0.25–1.32)	0.16
PUFAs (% of Energy)	4.27	5.70	7.10	8.85	
Model 1	1.00	0.86(0.40–1.83)	0.20(0.06–0.63)	0.60(0.26–1.36)	0.06
Model 2	1.00	0.66(0.28–1.55)	0.23(0.07–0.78)	0.52(0.21–1.29)	0.08
n-6 PUFAs (% of Energy)	3.64	4.90	6.20	7.90	
Model 1	1.00	0.86(0.40–1.83)	0.15(0.04–0.53)	0.66(0.30–1.44)	0.09
Model 2	1.00	0.68(0.29–1.58)	0.18(0.04–0.66)	0.55(0.22–1.33)	0.09
n-3 PUFAs (% of Energy)	0.30	0.42	0.54	0.70	
Model 1	1.00	1.53(0.67–3.48)	1.00(0.41–2.41)	0.80(0.31–2.02)	0.42
Model 2	1.00	1.38(0.56–3.42)	0.97(0.37–2.56)	0.74(0.27–2.02)	0.42
MUFA _(% of Energy)_	8.20	10.2	12.0	13.8	
Model 1	1.00	0.62(0.28–1.37)	[0.39(0.16–0.96)]	0.45(0.19–1.06)	0.03
Model 2	1.00	0.40(0.16–0.98)	0.40(0.15–1.08)	0.42(0.17–1.05)	0.05
Trans fatty acid _(% of Energy)_	1.04	1.72	2.43	3.50	
Model 1	1.00	0.38(0.15–0.96)	0.75(0.34–1.65)	0.65(0.28–1.47)	0.57
Model 2	1.00	0.42(0.15–1.18)	0.75(0.30–1.85)	0.76(0.30–1.94)	0.89
Cholesterol _(mg/day)_	124	143	158	188	
Model 1	1.00	3.25(1.13–9.31)	2.88(0.99–8.41)	3.07(1.06–8.86)	0.10
Model 2	1.00	2.96(0.94–9.29)	2.42(0.76–7.72)	3.39(1.04–10.9)	0.08
Total carbohydrate _(% of Energy)_	49.0	55.4	59.2	64.7	
Model 1	1.00	0.55(0.20–1.46)	0.90(0.38–2.15)	1.49(0.67–3.30)	0.23
Model 2	1.00	0.63(0.22–1.83)	0.83(0.31–2.22)	1.66(0.69–4.01)	0.20
Free sugars _(% of Energy)_	3.00	5.14	7.22	10.9	
Model 1	1.00	0.49(0.20–1.22)	1.15(0.54–2.46)	0.42(0.16–1.09)	0.21
Model 2	1.00	0.39(0.13–1.11)	1.33(0.57–3.13)	0.39(0.14–1.08)	0.24
Protein _(% of Energy)_	10.7	12.2	13.5	15.4	
Model 1	1.00	1.00(0.38–2.62)	1.91(0.80–4.55)	1.50(0.61–3.69)	0.22
Model 2	1.00	0.94(0.33–2.67)	1.67(0.65–4.28)	1.45(0.55–3.81)	0.31
Salt _(g/day)_	4.91	7.70	11.83	20.1	
Model 1	1.00	0.74(0.3–1.78)	1.08(0.48–2.44)	0.74(0.31–1.78)	0.67
Model 2	1.00	0.71(0.27–1.89)	0.96(0.36–2.55)	1.21(0.43–3.38)	0.51
Fruits and Vegetables _(g/day)_	236	420	624	999	
Model 1	1.00	1.20(0.51–2.83)	1.00(0.41–2.41)	1.10(0.46–2.62)	0.94
Model 2	1.00	1.10(0.43–2.84)	1.22(0.46–3.27)	1.39(0.46–4.13)	0.53
Total dietary fiber _(g/day)_	6.94	9.04	10.9	14.4	
Model 1	1.00	1.86(0.74–4.65)	1.56(0.61–3.99)	1.71(0.67–4.32)	0.39
Model 2	1.00	1.52(0.57–4.01)	1.17(0.41–3.27)	1.70(0.63–4.58)	0.37

SFA: Saturated fatty acid, PUFA: Poly unsaturated fatty acids

Model 1: Crude

Model 2: Adjusted for baseline age, sex, total energy intake, family history of diabetes, and BMI

Three major dietary patterns were identified; the healthy dietary pattern was highly loaded on vegetables, fruit and fruit juices, nuts, seeds, dairy and liquid oils; the unhealthy dietary pattern.

composed of fast foods, soft drinks, mayonnaise, salty snacks, tea, coffee, solid oils, sweets and sugar; the third dietary pattern, cereal/meat, was loaded densely on red and organ meat, fish and poultry, legumes, egg and refined grains **(**Table 4**)**.

**Table 4:** Dietary patterns identified in children and adolescents participating in the Tehran Lipid and Glucose Study

Food groups	Dietary patterns		
	Healthy	Unhealthy	Cereal/meat
Fast foods		0.43	
Soft drinks		0.55	
Mayonnaise	0.27	0.53	
Salty snacks	0.29	0.43	
Tea/coffee		0.49	
Solid oils		0.26	
Sweets and Sugar		0.20	
Red/organ meats	0.25	0.23	0.47
Fish and Poultry			0.26
Legumes			0.45
Egg			0.32
Potato			0.44
Whole grains			0.43
Refined grains			0.20
Liquid oils	0.35		0.21
Vegetables	0.51		0.22
Fruits	0.68		
Fruit juice	0.55		
Nuts and seeds	0.34		
High fat dairy	0.31		
Low fat dairy	0.23		
Variance (%)	9.96	8.22	7.45

Values are factor loadings of dietary patterns measured by factor analysis. Factor loadings below ±0.2 are not shown in the table for simplicity, eigenvalues> 1, Kaiser-Meyer-Olkin (KMO): 0.53

The odds ratios of developing MetS across quartiles of dietary pattern scores are presented in Table 5. There was no significant decreasing trend in the risk of MetS across quartiles of healthy and cereal/meat dietary patterns. A significant increased risk of MetS was observed in the highest quartile of unhealthy dietary pattern score compared to the lowest quartile after adjustment for confounding factors [OR: Q4:2.49 (CI:1.00–6.19); P trend = 0.03]; this association was not observed in the crude model. There was not any significant correlation between MetS components and three dietary pattern scores.

**Table 5:** Multivariable-adjusted odds ratio (95% CIs) for incident MetS according to quartiles of dietary pattern scores among 424 children and adolescents

		Quartile of Dietary pattern score			
	Q1	Q2	Q3	Q4	P _trend_*
Healthy (n)	106	106	106	106	
Model 1	1.00	0.37(0.14–0.93)	0.54(0.23–1.25)	0.73(0.33–1.59)	0.61
Model 2	1.00	0.36(0.13–0.99)	0.41(0.15–1.06)	0.70(0.28–1.72)	0.43
Unhealthy (n)	107	105	106	106	
Model 1	1.00	0.92(0.39–2.20)	0.64(0.25–1.65)	1.40(0.63–3.13)	0.42
Model 2	1.00	0.81(0.30–2.20)	0.93(0.33–2.57)	[2.49(1.00–6.19)]	0.03
Cereal/meat (n)	106	106	106	106	
Model 1	1.00	2.17(0.88–5.33)	1.71(0.67–4.32)	1.27(0.48–3.37)	0.88
Model 2	1.00	2.32(0.86–6.22)	1.54(0.56–4.28)	1.28(0.44–3.66)	0.91

Model 1: Crude

Model 2: Adjusted for baseline age, sex, total energy intake, family history of diabetes, and BMI

^*^Based on logistic regression model using median scores of dietary patterns in each quartile as a continuous variable

## Discussion

In our study, dietary patterns of Tehranian children and adolescents aged 6–19 years were identified and their dietary intakes were compared with WHO healthy diet recommendations. Also, the association of dietary adherence to WHO healthy diet components and dietary patterns with the risk of MetS and its components were assessed through 3.6 years of follow-up.

Compared with WHO recommendations, Iranian children and adolescents showed the highest adherence with cholesterol, free sugars, protein, total carbohydrate, fruit and vegetable, respectively; whereas, the lowest was observed for n-3 PUFAs, TFAs, SFAs and salt.

The mean dietary intake of total fat was higher than the upper limit of WHO healthy diet recommendation in both genders (32% vs 30% of energy); however, no correlation was seen between the risks of MetS across quartiles of total fat intake.

In our study population, n-3 PUFAs was consumed much lower than WHO recommendations. Previous studies showed that, dietary n-3 PUFA had a protective effect against MetS and low-grade inflammation among children and adolescents [23]; this was in contrast to our results, which seems to be due to the lower consumption of foods containing n-3 PUFA in the Iranian society.

Dietary SFAs up to 12% of energy showed a protective effect on MetS up to 80%; which disappeared in higher consumption of SFA. Although we could not find a study on children and adolescent regarding the correlation of SFA and Mets, but the present study was in line with Dehghan et al’s study which reported an inverse association between SFA intake and the risk of stroke in adults [24]. Replacing carbohydrate by SFA is also associated with a decreased risk of stroke by 20% among the Asian population. Higher carbohydrate, especially from refined sources and lower fat consumption is more common in low and middle income countries which have been shown to increase the metabolic risk factors. Individuals with high carbohydrate intake might benefit from a reduction in carbohydrate intake and increase in the consumption of fats [24, 25]. Thus the effect of SFA on MetS may also be affected by the remaining components of dietary macronutrients [26, 27].

In the present study, there was no correlation between higher intake of TFA (2 fold higher than WHO recommendation) and the risk of MetS or its components among children and adolescents. There were no studies showing the correlation between TFA and Mets among children and adolescent. Our results are inconsistent with the recent study claiming that there was a positive correlation between TFA and MetS among US adults [28].

In the present study, total dietary fiber, fruits and vegetables were received more than WHO recommendations, and may be considered as a factor in suppressing the adverse effects of SFA and TFA rich foods. There are limited data about the association of fruit and vegetable consumption with Mets among children and adolescents. Based on a previous study on 131 Latino children, an inverse association with total dietary fiber, particularly soluble fiber and MetS was found [29]. One study showed that the frequency of vegetable consumption in childhood is inversely associated with MetS in adulthood [30].

Salt intake among Iranian children and adolescents was 2 fold higher than WHO recommendation; however, there was no correlation between salt intake and the risk of MetS or hypertension in our study population. There are limited studies that have directly assessed the relationship between salt intake and the risk of MetS among children; but, a recent Korean study of 1738 boys aged 10–18 years revealed that high sodium intake may be independently associated with MetS [31]. In addition, previous studies showed that sodium intake more than WHO recommendations is positively associated with systolic BP and risk of pre-high BP and high BP, and this association may be stronger among those who are overweight or obese [32, 33]. There is strong evidence on the association between salt intake and high BP in children, adolescents and adults; however, some studies among children did not find an association between salt intake and hypertension, which was in line with our findings. These studies hypothesized that salt intake in childhood might lead to high BP in adulthood and older ages [33, 34]. Nonetheless, such a high rate of children and adolescents consuming salt more than recommended is worrisome, and individuals should minimize their salt intake by reducing processed foods and salty snacks, thus, contributing to healthier dietary patterns.

The results of our study can be interpreted in several aspects, MUFA intake showed a negative correlation with the risk of MetS up to 10–12% of energy intake. This association was weaker after adjustment of confounding factors. Further analysis in age and sex subgroups is needed to find out its moderators; however, total dietary fat may modulate this association, which emphasize that both the quality and quantity of dietary fats are relevant with Mets [9].

High intake of MUFA may suppress the adverse effect of expression of inflammatory genes that induce insulin resistance and secretion of inflammatory cytokines due to the rich SFA and TFA rich foods [35, 36]. To confirm this hypothesis, previous studies reported that there was negative association between the Mediterranean diet, rich in MUFA, and MetS among children and adolescents [37, 38].

Furthermore, in our study cholesterol and free sugar intake had the highest consistency with WHO recommendations. A study on 151 white girls showed that, sweetened beverages were the only dietary component related to MetS [39]. Another study of 424 subjects aged 6–18 years showed that high intakes of carbonated beverages increased the risk of MetS [7]. A recent CASPIAN-V study on 3843 Iranian children and adolescents showed that sweet dietary pattern increased the risk of MetS and some components [40]. Also, in the Framingham Heart Study of 6842 metabolically healthy adults, individuals consuming sugar sweetened beverages were more likely to display metabolic abnormalities compared to those who did not [41]. Therefore, high consistency of free sugar intake (> 80%) with WHO recommendations may be considered as a reason for preventing the incidence of MetS, despite high intake of SFA and TFA.

Due to in vivo interaction of macronutrients on body health, dietary components alone cannot be a good indicator for evaluating the incidence of MetS. Focusing on dietary patterns and considering all aspects of an individual’s dietary intake provides more precision to determine the risk of MetS among children and adolescents. The present study supports the positive correlation between unhealthy diet and MetS. Based on previous studies, the ‘Western’ dietary pattern was associated with a greater risk for MetS among children and adolescent [35, 42, 43]. Also there was no significant association between unhealthy dietary pattern and MetS in the crude model due to different relationships in age and sex groups or BMI and energy intake levels with MetS; after controlling these confounding variables, this association became significant.

Our study has its limitations; using an FFQ can estimate the usual intake of participants not actual intake, however, this FFQ can rank individuals accurately based on their intakes. The FFQ relies on recalling of food consumption, which is difficult in children; although, using experienced interviewers helps subjects or their parents to remind what they eat regarding the quality and quantity of food items, which decrease memory limitation. Lack of information on puberty stage and Carotid intima-media thickness (IMT) are other limitations of our study. It is evident that puberty stage can affect MetS components. IMT helps in predicting childhood and adolescent MetS more precisely [44]. Also socio-economic status of the child/adolescent’s family may play a role in the risk of MetS, which was not assessed in our study. Loss to follow up occurred in 20% of our study participants which may raise the selection bias. Moreover, lack of a significant relationship between WHO healthy diet components and MetS may be due to the limited number of MetS subjects in each quartile of these food or nutrient items.

Our study has major strengths too, to our knowledge, this is the first study assessing the adherence to nutritional intake based on WHO healthy diet, in a large nationally representative sample of Iranian children and adolescents aged 6–19 years; also, it has a prospective design with an appropriate follow-up duration for assessing the incidence of MetS. In addition, national cut-off points were used for assessing abdominal obesity in children and adolescents. Measurement of confounding factors is the other strength of our study.

## Conclusion

Our results demonstrated that the majority of our study population did not meet WHO healthy diet recommendations on n-3 PUFAs, TFAs and salt intake; also, WHO healthy diet alone cannot predict the risk of MetS and its components in Tehranian children and adolescents. Adherence to unhealthy dietary pattern was associated with two-fold increase in MetS risk; thus, reducing the consumption of unhealthy food items including fast foods, sweetened beverages, salty snacks, sweets and high fat red meats may reduce the risk of MetS in children and adolescents.

## Data Availability

The datasets generated and/or analyzed during the current study are not publicly available, but are available from the corresponding author on reasonable request.

## Abbreviations

BMI: Body mass index
BP: Blood Pressure
FBS: Fasting blood sugar
FCT: Food composition table
FFQ: Food frequency questionnaire
HDL-C: High density lipoprotein cholesterol
IMT: Carotid intima-media thickness
MAQ: Modifiable activity questionnaire
MET: Metabolic equivalent
MetS: Metabolic syndrome
MUFA: Mono-unsaturated fatty acid
PUFA: Poly-unsaturated fatty acid
SFA: Saturated fatty acids
TFA: Trans-fatty acids
TG: Triglycerides
TLGS: Tehran Lipid and Glucose Study
WC: Waist circumference
WHO: World Health Organization

## References

[1] Grundy SM (2016). Metabolic syndrome update. Trends Cardiovasc Med.

[2] Asghari G, Yuzbashian E, Mirmiran P, Mahmoodi B, Azizi F (2015). Fast food intake increases the incidence of metabolic syndrome in children and adolescents: Tehran lipid and glucose study. PLoS One.

[3] Asghari G, Yuzbashian E, Mirmiran P, Hooshmand F, Najafi R, Azizi F (2016). Dietary approaches to stop hypertension (DASH) dietary pattern is associated with reduced incidence of metabolic syndrome in children and adolescents. J Pediatr.

[4] Olson M, Chambers M, Shaibi G (2017). Pediatric markers of adult cardiovascular disease. Curr Pediatr Rev.

[5] Kotecha PV, Patel SV, Baxi RK, Mazumdar VS, Shobha M, Mehta KG (2013). Dietary pattern of schoolgoing adolescents in urban Baroda, India. J Health Popul Nutr.

[6] Mikkila V, Rasanen L, Raitakari OT, Pietinen P, Viikari J (2005). Consistent dietary patterns identified from childhood to adulthood: the cardiovascular risk in young Finns study. Br J Nutr.

[7] Mirmiran P, Yuzbashian E, Asghari G, Hosseinpour-Niazi S, Azizi F (2015). Consumption of sugar sweetened beverage is associated with incidence of metabolic syndrome in Tehranian children and adolescents. Nutr Metab (Lond).

[8] WHO. FACT SHEET N°394, Healthy diet. 2015.

[9] Hosseinpour-Niazi S, Mirmiran P, Fallah-ghohroudi A, Azizi F (2015). Combined effect of unsaturated fatty acids and saturated fatty acids on the metabolic syndrome: Tehran lipid and glucose study. J Health Popul Nutr.

[10] Asghari G, Yuzbashian E, Mirmiran P, Bahadoran Z, Azizi F (2016). Prediction of metabolic syndrome by a high intake of energy-dense nutrient-poor snacks in Iranian children and adolescents. Pediatr Res.

[11] Diet, nutrition and the prevention of chronic diseases. World Health Organization technical report series. 2003;916:i-viii, 1–149, backcover. 12768890

[12] Mohseni-Takalloo Sahar, Hosseini-Esfahani Firoozeh, Mirmiran Parvin, Azizi Fereidoun (2016). Associations of Pre-Defined Dietary Patterns with Obesity Associated Phenotypes in Tehranian Adolescents. Nutrients.

[13] Azizi F, Ghanbarian A, Momenan AA, Hadaegh F, Mirmiran P, Hedayati M (2009). Prevention of non-communicable disease in a population in nutrition transition: Tehran lipid and glucose study phase II. Trials.

[14] Esfahani FH, Asghari G, Mirmiran P, Azizi F (2010). Reproducibility and relative validity of food group intake in a food frequency questionnaire developed for the Tehran lipid and glucose study. J Epidemiol.

[15] Mirmiran P, Esfahani FH, Mehrabi Y, Hedayati M, Azizi F (2010). Reliability and relative validity of an FFQ for nutrients in the Tehran lipid and glucose study. Public Health Nutr.

[16] USDA. Food composition table (FCT). 2010.

[17] Rad AH, Esmaeili M, Abdollahi M, Azar M (2007). Compiling and validation of Iranian food composition tables. Ann Nutr Metab.

[18] Ainsworth BE, Haskell WL, Whitt MC, Irwin ML, Swartz AM, Strath SJ (2000). Compendium of physical activities: an update of activity codes and MET intensities. Med Sci Sports Exerc.

[19] Delshad M, Ghanbarian A, Ghaleh NR, Amirshekari G, Askari S, Azizi F (2015). Reliability and validity of the modifiable activity questionnaire for an Iranian urban adolescent population. Int J Prev Med.

[20] Cook S, Weitzman M, Auinger P, Nguyen M, Dietz WH (2003). Prevalence of a metabolic syndrome phenotype in adolescents: findings from the third National Health and nutrition examination survey, 1988-1994. Arch Pediatr Adolesc Med.

[21] Kelishadi R, Gouya MM, Ardalan G, Hosseini M, Motaghian M, Delavari A (2007). First reference curves of waist and hip circumferences in an Asian population of youths: CASPIAN study. J Trop Pediatr.

[22] The fourth report on the diagnosis, evaluation, and treatment of high blood pressure in children and adolescents. Pediatrics. 2004;114:555–576. 15286277

[23] Pacifico L, Giansanti S, Gallozzi A, Chiesa C (2014). Long chain omega-3 polyunsaturated fatty acids in pediatric metabolic syndrome. Mini Rev Med Chem.

[24] Dehghan M, Mente A, Zhang X, Swaminathan S, Li W, Mohan V (2017). Associations of fats and carbohydrate intake with cardiovascular disease and mortality in 18 countries from five continents (PURE): a prospective cohort study. Lancet (London, England).

[25] Yu D, Shu XO, Li H, Xiang YB, Yang G, Gao YT (2013). Dietary carbohydrates, refined grains, glycemic load, and risk of coronary heart disease in Chinese adults. Am J Epidemiol.

[26] Wan Y, Wang F, Yuan J, Li J, Jiang D, Zhang J (2017). Effects of macronutrient distribution on weight and related Cardiometabolic profile in healthy non-obese Chinese: a 6-month Randomized Controlled-Feeding Trial. EBioMedicine.

[27] Gow ML, Ho M, Burrows TL, Baur LA, Stewart L, Hutchesson MJ (2014). Impact of dietary macronutrient distribution on BMI and cardiometabolic outcomes in overweight and obese children and adolescents: a systematic review. Nutr Rev.

[28] Zhang Z, Gillespie C, Yang Q (2017). Plasma trans-fatty acid concentrations continue to be associated with metabolic syndrome among US adults after reductions in trans-fatty acid intake. Nutrition research (New York, NY).

[29] Ventura EE, Davis JN, Alexander KE, Shaibi GQ, Lee W, Byrd-Williams CE (2008). Dietary intake and the metabolic syndrome in overweight Latino children. J Am Diet Assoc.

[30] Jaaskelainen P, Magnussen CG, Pahkala K, Mikkila V, Kahonen M, Sabin MA (2012). Childhood nutrition in predicting metabolic syndrome in adults: the cardiovascular risk in young Finns study. Diabetes Care.

[31] So CH, Jeong HR, Shim YS. Association of the urinary sodium to urinary specific gravity ratio with metabolic syndrome in Korean children and adolescents : The Korea National Health and Nutrition Examination Survey 2010–2013. PLoS One. 2017:12:e0189934. 10.1371/journal.pone.0189934 PMC5734790 29253859

[32] Yang Q, Zhang Z, Kuklina EV, Fang J, Ayala C, Hong Y (2012). Sodium intake and blood pressure among US children and adolescents. Pediatrics..

[33] Correia-Costa L, Cosme D, Nogueira-Silva L, Morato M, Sousa T, Moura C (2016). Gender and obesity modify the impact of salt intake on blood pressure in children. Pediatric Nephrol (Berlin, Germany).

[34] Shi L, Krupp D, Remer T (2014). Salt, fruit and vegetable consumption and blood pressure development: a longitudinal investigation in healthy children. Br J Nutr.

[35] Taghizadeh S, Alizadeh M. The Role of Lipids in the Pathogenesis of Metabolic Syndrome in Adolescents. Experimental and clinical endocrinology & diabetes : official journal, German Society of Endocrinology [and] German Diabetes Association. 2018;126:14–22. 10.1055/s-0043-106439 29117624

[36] van Dijk SJ, Feskens EJ, Bos MB, Hoelen DW, Heijligenberg R, Bromhaar MG (2009). A saturated fatty acid-rich diet induces an obesity-linked proinflammatory gene expression profile in adipose tissue of subjects at risk of metabolic syndrome. Am J Clin Nutr.

[37] Martin-Calvo N, Chavarro JE, Falbe J, Hu FB, Field AE (2016). Adherence to the Mediterranean dietary pattern and BMI change among US adolescents. Int J Obesity (2005).

[38] Velazquez-Lopez L, Santiago-Diaz G, Nava-Hernandez J, Munoz-Torres AV, Medina-Bravo P, Torres-Tamayo M (2014). Mediterranean-style diet reduces metabolic syndrome components in obese children and adolescents with obesity. BMC Pediatr.

[39] Ventura AK, Loken E, Birch LL (2006). Risk profiles for metabolic syndrome in a nonclinical sample of adolescent girls. Pediatrics..

[40] Kelishadi R, Heshmat R, Mansourian M, Motlagh ME, Ziaodini H, Taheri M (2018). Association of dietary patterns with continuous metabolic syndrome in children and adolescents; a nationwide propensity score-matched analysis: the CASPIAN-V study. Diabetol Metab Syndr.

[41] Green AK, Jacques PF, Rogers G, Fox CS, Meigs JB, McKeown NM (2014). Sugar-sweetened beverages and prevalence of the metabolically abnormal phenotype in the Framingham Heart Study. Obesity (Silver Spring, Md).

[42] Ambrosini GL, Huang RC, Mori TA, Hands BP, O'Sullivan TA, de Klerk NH (2010). Dietary patterns and markers for the metabolic syndrome in Australian adolescents. Nutr Metab Cardiovasc Dis.

[43] Hosseini-Esfahani F, Hosseinpour-Niazi S, Asghari G, Bahadoran Z, Moslehi N, Golzarand M (2018). Nutrition and cardio-metabolic risk factors: findings from 20 years of the Tehran lipid and glucose study. Int J Endocrinol Metab.

[44] Juonala M, Singh GR, Davison B, van Schilfgaarde K, Skilton MR, Sabin MA (2016). Childhood metabolic syndrome, inflammation and carotid intima-media thickness. The aboriginal birth cohort study. Int J Cardiol.

